# Studying the UK job market during the COVID-19 crisis with online job ads

**DOI:** 10.1371/journal.pone.0251431

**Published:** 2021-05-27

**Authors:** Rudy Arthur

**Affiliations:** Department of Computer Science, University of Exeter, Exeter, United Kingdom; Nanyang Technological University, SINGAPORE

## Abstract

The COVID-19 global pandemic and the lockdown policies enacted to mitigate it have had profound effects on the labour market. Understanding these effects requires us to obtain and analyse data in as close to real time as possible, especially as rules change rapidly and local lockdowns are enacted. This work studies the UK labour market by analysing data from the online job board Reed.co.uk, using topic modelling and geo-inference methods to break down the data by sector and geography. I also study how the salary, contract type, and mode of work have changed since the COVID-19 crisis hit the UK in March. Overall, vacancies were down by 60 to 70% in the first weeks of lockdown. By the end of the year numbers had recovered somewhat, but the total job ad deficit is measured to be over 40%. Broken down by sector, vacancies for hospitality and graduate jobs are greatly reduced, while there were more care work and nursing vacancies during lockdown. Differences by geography are less significant than between sectors, though there is some indication that local lockdowns stall recovery and less badly hit areas may have experienced a smaller reduction in vacancies. There are also small but significant changes in the salary distribution and number of full time and permanent jobs. As well as the analysis, this work presents an open methodology that enables a rapid and detailed survey of the job market in unsettled conditions and describes a web application jobtrender.com that allows others to query this data set.

## Introduction

The COVID-19 pandemic claimed over 90,000 lives in the UK as of the January, 2021 [1]. The most drastic measure to limit the spread of COVID-19 has been the imposition of so-called lockdown measures. ‘Lockdown’ refers to national or regional orders calling for the closure of businesses and restriction of assembly and travel. Lockdown policies have not been uniform across nations. Some areas, for example Sweden and Japan, implemented essentially voluntary measures [2, 3] while others, for example China and Germany, imposed and enforced quite severe restrictions on assembly and business opening [4, 5].

The UK’s lockdown policy was between these two extremes. Beginning somewhat later than many other European nations, on March 21st, 2020 the UK government introduced The Health Protection (Coronavirus, Business Closure) (England) Regulations 2020 [6] which was superseded by the The Health Protection (Coronavirus, Restrictions) (England) Regulations 2020 on March 26th [7]. This piece of legislation, which will hereafter be referred to as ‘lockdown’, included restricted freedom of movement, bans on gatherings, and enforced business closures. The lifting of some of these rules began on May 13th, though in some heavily affected areas stricter measures were retained or re-imposed [8, 9] and the rules have been subsequently modified, e.g., ‘the rule of six’ [10], the ‘tier system’, and other national lockdowns [11].

The economic impacts of the COVID-19 crisis have been severe. The UK saw a 125.9% increase in unemployment claims between March and May and vacancies dropping by 58% over the same period [12]. Headline unemployment numbers did not immediately rise [13], but by the end of the year the unemployment rate was on a markedly upward trend, standing at 4.9% as of December 2020 [14]. To provide support during this period the UK government introduced unprecedented measures: namely the Coronavirus Job Retention (furlough) Scheme [15] to attempt to keep unemployment rates in check by providing grants to businesses to pay up to 80% of worker salaries.

The academic study of lockdown has necessarily been reactive and observational. The rapid onset of the crisis has meant that researchers have had to source and analyse real time labour market information. For example Bick et al. [16] use an online labour market survey; Chetty et al. [17] use anonymised data from several large companies; Hensvik et al. [18] use vacancy postings while Forsythe et al. [19] use data from the job market analysis company Burning Glass (https://www.burning-glass.com/). Surveys have also played an important role. Again in the US, Coibion et al. [20] use a large scale household survey and Bartik et al. [21] surveyed small business owners.

Different methodologies yield useful insights into the effect of the COVID-19 crisis on the labour market, illuminating a different aspects of the same picture. The job market in the US has probably been the most extensively studied. For example Forsythe et al. [19] show a 44% drop in vacancy postings between February and April in the US, observed across occupational categories (essential or non-essential work) as well as across states which may have had different lockdown policies. Bick et al. [16] report similar drops in the US across sector and demographics during the same period, with a slow recovery in the months after. The survey methodology of Coibion et al. [20] confirms that not only are job losses greater than measured from unemployment claims, jobseekers stopped actively searching for employment. Bartik et al. found employee counts reduced by 40% at the start of the crisis in April 2020, with many of the small businesses surveyed very financially fragile.

The European job market has been less extensively researched, however some studies exist. For example, analysis of vacancy postings in Sweden, which has had probably the least restrictive lockdown in Europe, shows a drop in job adverts by around 40% [18]. Hensvik et al. also report that job seekers are searching less intensively and redirecting their searches towards less severely hit occupations.

This work is concerned with the UK labour market and adds to the body of knowledge about how lockdown policy affected job vacancies in that country. The most directly similar work to this is Costa et al. [22] who study two months of 2020 job vacancy data from the UK government’s ‘find-a-job’ website (https://www.gov.uk/find-a-job). They find significant reductions in adverts across sectors, wage bands, and geographies, with an uneven recovery centered on the health and social care sector in more affluent areas. The longer time series studied here allows a more detailed study of the recovery, as well as the potential to identify additional effects due to changing rules and new lockdowns. There is also analysis by the large job board Indeed [23] which shows a significant reduction in job vacancy postings. These findings are somewhat scattered and piecemeal but broadly agree with what is observed on Reed.co.uk.

The effect of the crisis on different sectors of the economy has exacerbated previously existing inequalities [24]. In Europe Adams-Prassl et al. [25] found that workers in Germany were insulated from job losses by longstanding institutional frameworks, compared to workers in the UK who are in a much more precarious position. The same work also finds that job losses and reductions in earnings disproportionately affect women, workers without a university degree, and younger people.

There is still serious academic debate about the efficacy of lockdown as a disease control measure [26, 27], how it should be implemented [28], and the negative and unintended consequences of these policies on health outcomes [29]. This work is an observational study of the effect of the effect of COVID-19, lockdown, and other disease control measures on the job market in the UK. Topic modelling and location inference methods will enable cross sectioning the job vacancies data. The technical approach is similar to that used by Burning Glass or [30]. In [30] Thurgood et al. used an unsupervised machine learning method on a corpus of online job adverts to discover how the labour market is segmented. This work uses a similar data set, sourced from the same online job board, but applies a new supervised method which gives more robust results and adds a location inference step to enable geographic analysis.

This work confirms the observations of many others on the negative impact of lockdown on the UK labour market using a novel data source. This data set contains around nine months of ‘post crash’ data which I break down by sector and geography. Issues of accessibility are crucial to allow interested, but non-expert, parties the ability to query the job data, e.g., a town or local council. To facilitate this, I have also developed a web application where this data set can be queried through a dashboard interface.

The specific findings of this work are of a widespread and significant drop in UK job vacancies, heavily concentrated in some sectors (hospitality, tourism) and spread roughly evenly across geography and the salary scale, in agreement with [22]. After the initial drop, the recovery in posts has been steady with many sectors and areas returning to close to levels observed in early 2019 by the end of the study period. While the results are not themselves surprising, this paper presents a new and useful methodology for researchers, utilising a novel and open data source together with new topic modelling and geographic inference methods. For users of labour market information, the web interface makes this data accessible to anyone looking to understand the ‘live’ job market in the UK.

## Data and methods

The online job board Reed.co.uk is used here. Reed is a large recruitment agency and owner of the UK’s first recruitment website, which receives around 7 million visits per month (https://www.reed.co.uk/about). Aside from its popularity, what makes it suitable for this analysis is an Application Programmer Interface (API) (https://www.reed.co.uk/developers) which allows downloading job adverts.

Each job advert on Reed has a unique, sequential index number. Starting at index 37000000, with the first full day of collection January 11th, 2019, two full years of data, up to January 11th, 2021, were collected, totalling around 4.7 million job ads. This should be sufficient to establish a baseline of vacancy information before COVID-19 became a national issue, observe the entire first lockdown period as well as a long enough time-frame to study any potential recovery. With only two years of data it is difficult to observe long term trends due to Brexit or other social, political, or economic forces, for that I rely on official statistics e.g. [14].

**Fig 1:**
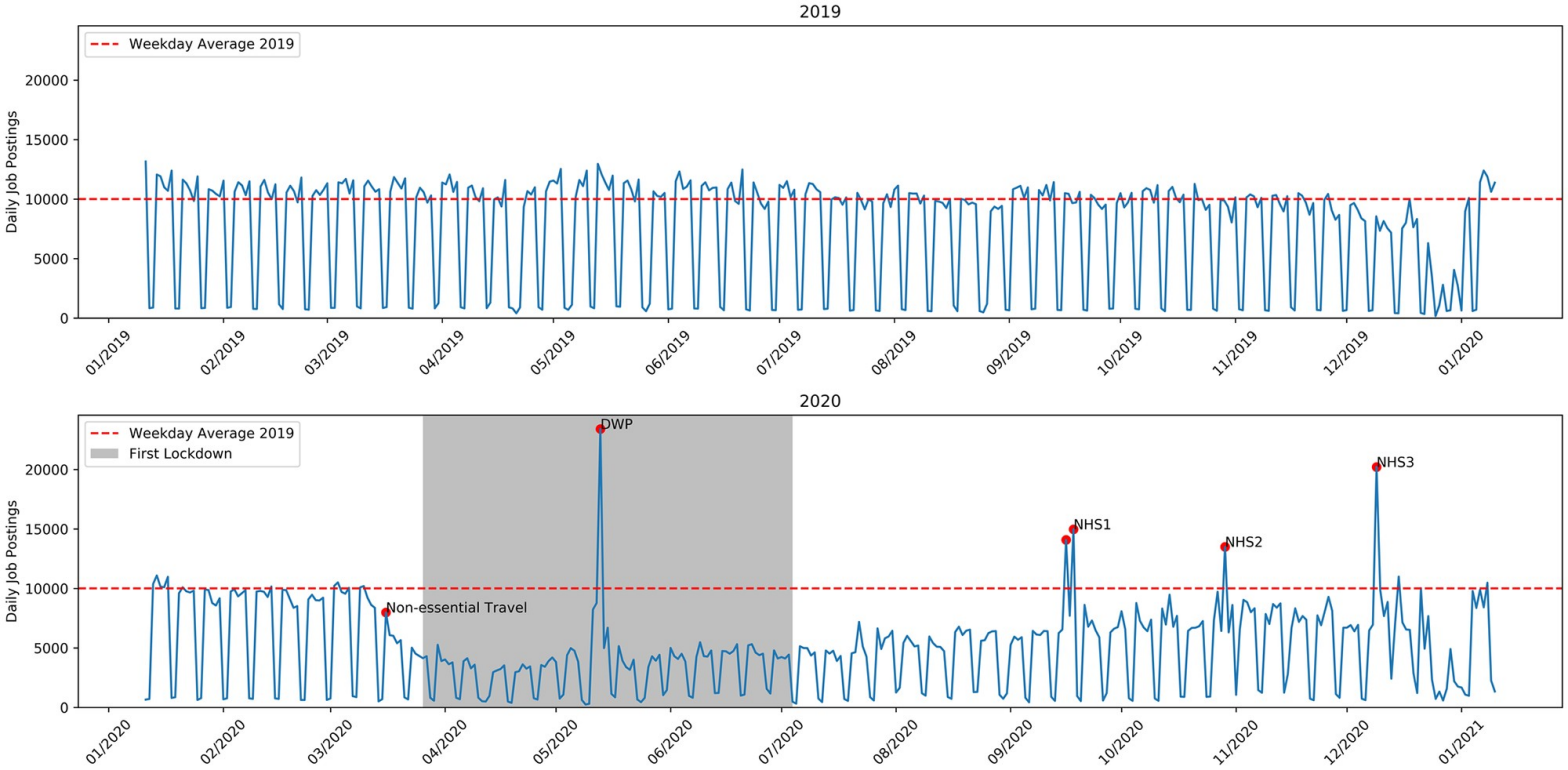
Daily job postings on Reed over the study period. The periodic dips correspond to weekends and public holidays. Some key dates are indicated and discussed in the text.

Fig 1 shows the number of job adverts collected per day. Job adverts which have been deleted or removed are returned by the API as Javascript Object Notation (JSON) objects [31] with all null values and are not included in Fig 1. There is no indication that these removed job ads have a significant impact and they represent only around 3% of the total number of records, spread fairly uniformly across the period.

Fig 1 shows some trends quite clearly. Key events are indicated on the plot, as well as the period I have defined as ‘lockdown’. The 2019 data shows a remarkably steady number of daily job ad postings throughout the year, however there is a distinct downward trend beginning in late November/early December. The first recorded case of coronavirus in the UK on the January 31st, 2020 is not associated with any change in trend. At this point coronavirus was not considered to be a major issue in the UK. A distinct drop in the number of job ads posted starts on March 16th, 2020 when UK Prime Minister Boris Johnson issued advice against “non-essential” travel and contact. Voluntary measures were in place before this, as well as press rumours of the soon to be announced lockdown, and the data indicates the start of the drop in job vacancies preceded the announcement. By the time lockdown began the number of ads was reduced to under half of the early year baseline.

A major spike in job ads, labelled DWP, occurs on the same day as the second amendment to the Health Protection Act [7], May 13th, 2020, which allowed for the re-opening of certain businesses and services e.g. garden centres, tennis courts and recycling centres. However it is not the case that this spike was caused by the announcement, as the vast majority of these jobs were posted by the UK’s Department of Work and Pensions. This body, among other things, manages the UK’s welfare system and maintains its own job advertisement board, findajob.dwp.gov.uk. On this day roughly 20000 job ads of all kinds were cross-posted to Reed from this site. The other spikes labelled NHS1, NHS2, and NHS3 also correspond to a sudden surge in job advertisements from government agencies. The NHS spikes represent between 8000 and 14000, mostly nursing, jobs posted by the NHS Business Services Authority on the corresponding days. This agency also has its own job board, jobs.nhs.uk, and these are likely cross postings designed to give their adverts a wider reach.

**Fig 2:**
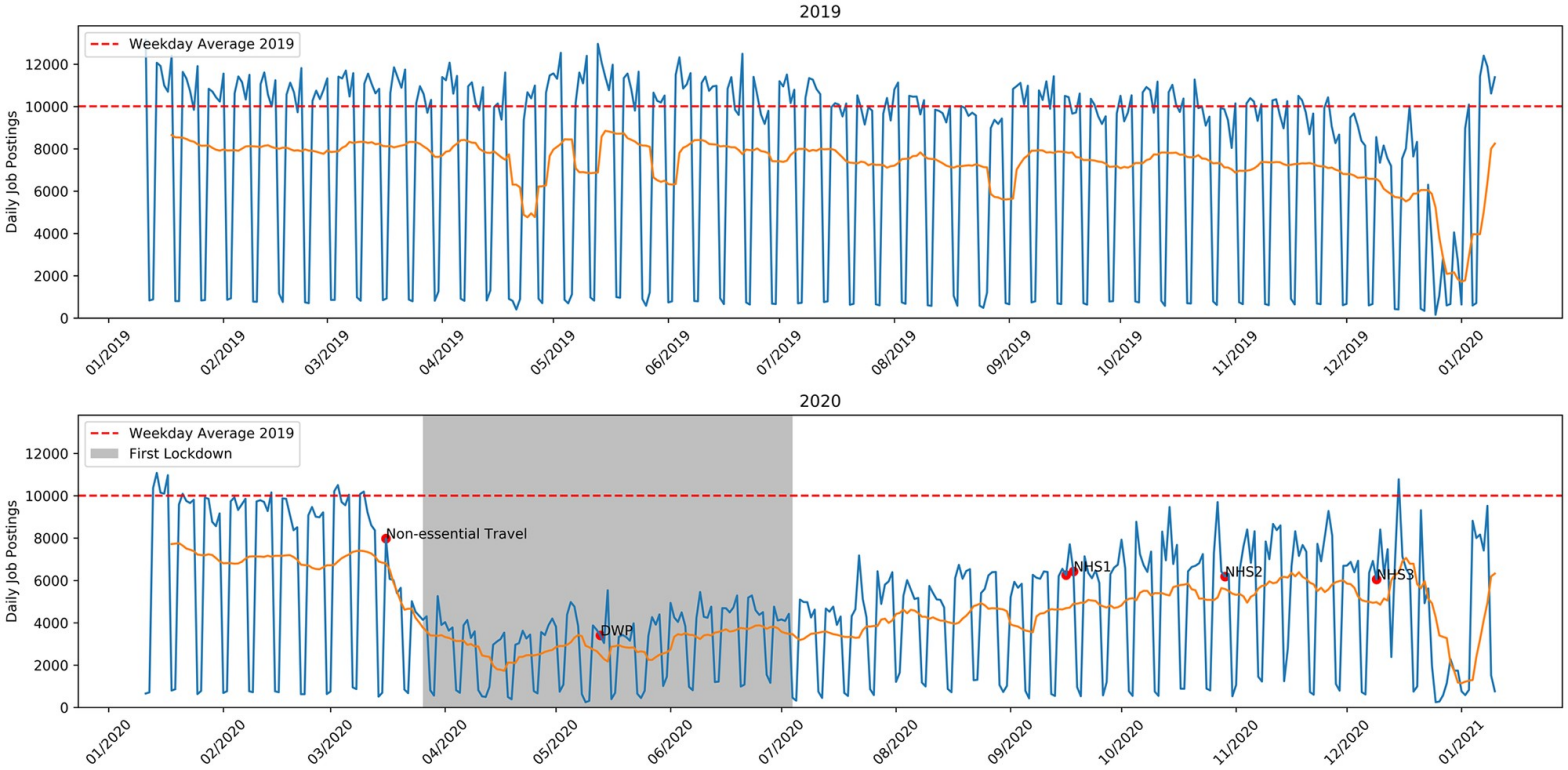
Daily job postings with the cross posts from the two other major job boards removed. Solid orange line is the 7 day running average count.

In light of this, I removed jobs posted by these two agencies from the data set. Specifically, I remove all ads posted by employers ‘DWP Teaching’, ‘Department of Work & Pensions’ and ‘NHS Business Services Authority’. The weekday/weekend variation is smoothed by showing the 7 day running averages. The resulting time series is shown in Fig 2. With the spikes removed it is clear that the number of jobs posted per day rapidly fell in March but has since slowly grown from its nadir in mid-April. As of January 2021 the number of posts per day seems to have recovered to pre-crisis levels. The total number of job ads posted in the 2019 period was 2691648 compared to 1700687 in the 2020 period, a deficit of 990961 or 37% fewer job adverts. If March 16th, 2020 is taken as the date on which the job market began to react to the crisis, comparing the period from March 16th to Jan 10th in 2019 and 2020 gives 2166551 and 1245180 job ads in 2019 compared to 2020, or a deficit of 42.5%.

### Topic modelling

Though the Reed website is searchable by topic and sector, the JSON payload returned by Reed’s API does not include a theme or topic marker and so this must be inferred. Algorithms such as Latent Dirichlet Allocation (LDA) [32] and Doc2Vec [33] transform text documents to low dimensional vector representations which enhance automatic topic detection algorithms. LDA has previously been used with success on a very similar data set [30].

I attempted to perform unsupervised clustering using LDA and Doc2Vec. While some categories of job are readily detectable with unsupervised methods, e.g., software developer, the topics or clusters detected are often not stable when varying the algorithm parameters. Measures like coherence [34] fail to provide obvious evidence in favour of any one parameter set and, in particular, for an optimal number of topics. A second reason to avoid unsupervised methods is that I want to study predetermined job categories e.g. nursing, teaching, or graduate jobs, to observe the reaction of these sectors to the COVID crisis. Thus the desired labels are known and it should be possible to obtain better results by providing more information to the classification algorithm. I therefore take a different and somewhat simpler approach than [30] which nevertheless suffices to identify topics.

For each ad I combine the job title with the job description to constitute a ‘document’. The document text is cleaned to remove HTML artefacts, lower-cased, lemmatised using the wordnet lemmatiser [35] and tokenised. Data from 2019 (Reed job ids 39500000 to 39600000) is used to collect a number of ‘seed’ documents. These seeds are collections of job ads which are representative of given sectors *S*_*c*_ = {*d*_*c*1_, *d*_*c*2_, …, *d*_*cn*_}. The label *c* is a job category, e.g., ‘teacher’ and the documents *d*_*ci*_ are job adverts definitively in that sector. These were determined by searching for common job titles in each category, for example, in ‘retail’ these are jobs with the terms ‘order picker’, ‘shop supervisor’, ‘retail assistant’ or ‘picker packer’ in the job title. The full list of categories is given in Table 1 and the job list used to build the seeds is available in the linked git repository: https://github.com/rudyarthur/COVIDJobAds. These categories were obtained by manual inspection of the data as well as comparison with the browsable job areas on the main Reed website.

**Table 1:** Job category labels. These labels are summaries of the most common job title in each category. The category ‘other’ contains jobs which could not be classified into one of the other categories.

account	accountant	administrator	assistant
business	buyer	care	charity
cleaner	construction	customer	data
delivery	electrician	finance	garage
graduate	hgv	hotel	hr
itsupportengineer	kitchen	machine	marketing
nurse	nursery	physio	plumber
prison	production	productionmanager	project
property	receptionist	recruitment	retail
sale	security	server	software
solicitor	storemanager	support	surveyor
teacher	vehicle	warehouse	welsh
other			

To get examples of the ‘other’ category I used Gensim’s implementation of TF-IDF [36] to transform each document into a vector. I then compute the mean cosine similarity of each job with all the vectors in each seed set. If the job has a mean similarity score less than 0.04 (roughly the 5th percentile) with any of the categories it is used as an example of the ‘other’ category. In this way 2205 jobs are identified as not similar to any of the given classes and labelled as ‘other’. The number 0.04 was found to work well in practice, however the method is robust to variations of this threshold.

With the given seeds as training data I tried a number of classification methods, finding that a simple decision tree [37] preformed best, achieving a Subset Accuracy score of 0.922, a Balanced Accuracy of 0.884, and a Cohen’s Kappa of 0.919 on an 80/20 train/test split of the seed data. When 100000 different job ads are passed to the classifier 85% are classified into one of the named categories with 15% put into the ‘other’ class. Below we will show word clouds built from documents in a number of classes. These seem very reasonable and a manual inspection of the confusion matrix finds that mistakes are almost invariably confusion of two similar categories e.g. ‘accountant’ and ‘finance’. Table 2 shows the most common mis-classifications made by our algorithm.

**Table 2:** Out of 8844 examples in the test set 196 are misclassified. The most common confusion made by the decision tree seems to be between office finance roles e.g. ‘finance’ includes jobs like ‘finance assistant’ and ‘purchase ledger clerk’ which have similar requirements to ‘accountant’ jobs. Many of these jobs are classified similarly by Reed themselves, so the small number of classification errors are made on ambiguous ads, showing the strength of the method.

True	False	Count
garage	customer	5
sale	customer	5
finance	hr	5
account	accountant	5
business	sale	6
account	finance	8
administrator	finance	9
finance	accountant	26

### Location inference

**Fig 3:**
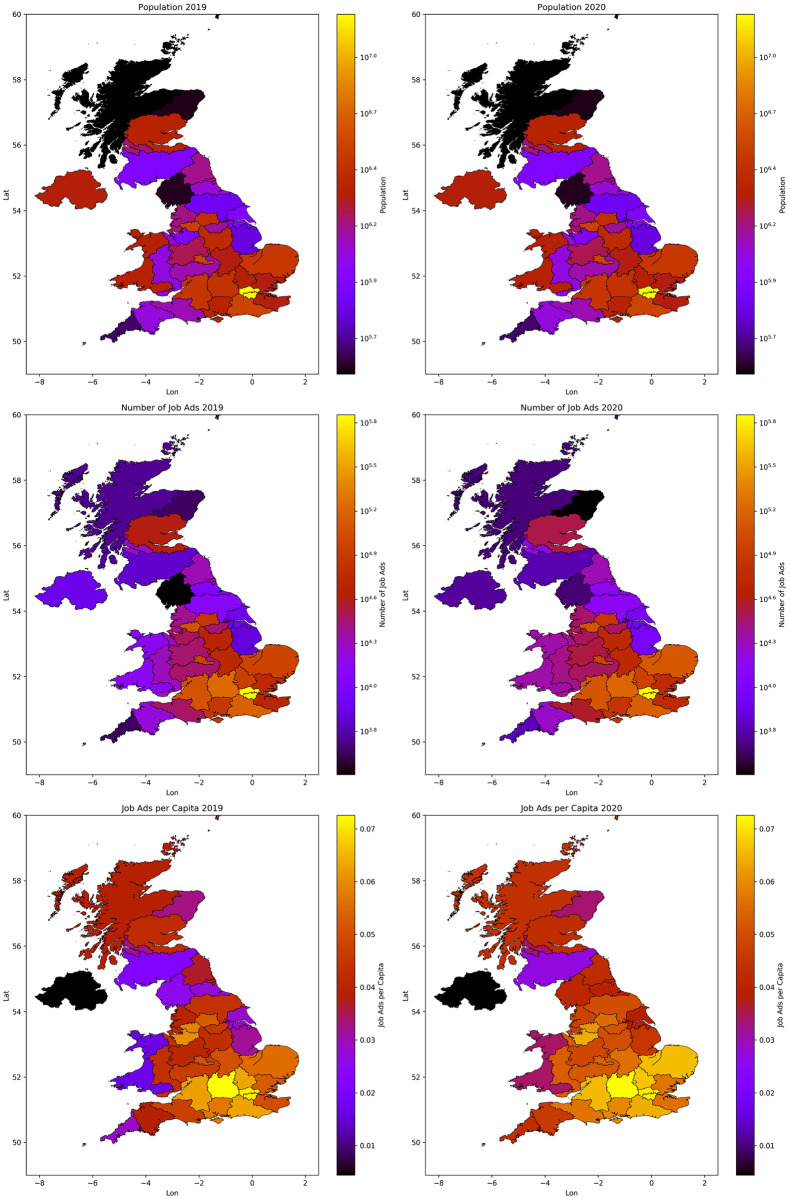
The administrative regions shown are the NUTS2 regions, except the London NUTS2 regions have been combined into ‘Greater London’. This is because a very large number of job ads give the location ‘London’, rather than a specific borough. The top row shows UK population, the middle shows the number of job ads posted and the bottom plot shows the number of job ads divided by the population of each region. The left column is 2019 and the right is 2020. Polygon boundary data is obtained from https://data.gov.uk.

Location inference is done using the ‘location’ field in the advert’s JSON. Some adverts are only localised at the county level, e.g., Devon. These are identified by checking against a list of UK administrative counties. The rest of the adverts are checked by querying the location field against the Geonames [38] and Nominatim databases [39], in that order. Geonames is effective at returning co-ordinates for larger towns and cities [40]. Nominatim is a web based geo-coding service which uses OpenStreetMap data to find locations by name and address. Since the same locations re-occur multiple times, every Nominatim lookup is saved in a database which is queried before calling the web service. This process returns GPS coordinates or bounding boxes for 97% of all non-null job adverts. Fig 3 shows the spatial distribution of job ads, localised to NUTS2 regions [41]. The geographic distribution of job adverts roughly corresponds to the UK population distribution [42]. The number of ads normalised by population (the bottom map in Fig 3), shows a higher density in the south east. There are also some changes from 2019 to 2020, despite over all fewer jobs, the per capita numbers are slightly higher in north east of England and rural areas of Cornwall and west Wales. The north/south divide is likely reflective of economic disparities within the UK [43]. However there are also potential differences in the popularity of the job board Reed.co.uk in different regions, or for different industries, e.g. it hosts very few farming jobs. Even without knowing how use of Reed is spread across the UK relative trends can still be studied. Some changes may reflect a drift in the user base of the site, but large and sudden changes are likely to be caused by significant exogenous events.

## Jobs by sector

This section presents a collection of time series for different job classes identified by topic modelling across the study period. These classes were chosen to highlight job categories which were likely to be impacted differently by the COVID-19 crisis. I expected delivery, nursing and care work demand to increase, while hospitality is expected to decrease. Software was chosen as a job which could in principle be done remotely so it would be interesting to see how this affected vacancy numbers. The number of teachers required should be largely unaffected by the crisis. I also look at graduate jobs. For clarity, note that in the UK the phrase ‘graduate job’ refers to jobs that specifically target recent university graduates and is not a generic term for jobs requiring a university degree. Graduate jobs are entry level positions, usually offered by large employers taking on a whole cohort of new employees as part of a ‘graduate scheme’ or program. Adams-Prassl et al. [25] report that people with a university degree were less likely to experience job loss, however as graduate schemes are highly focused on training and are offered to younger people, they might be postponed or cancelled in the face of uncertainty.

The job categories have been chosen to be representative of key sectors and to show interesting trends, they do not represent every job sector that can be found in the data. The word clouds only use jobs from the period March 16th to Jan 10th in both years. This is to attempt to detect any change in common job titles induced by COVID.

**Fig 4:**
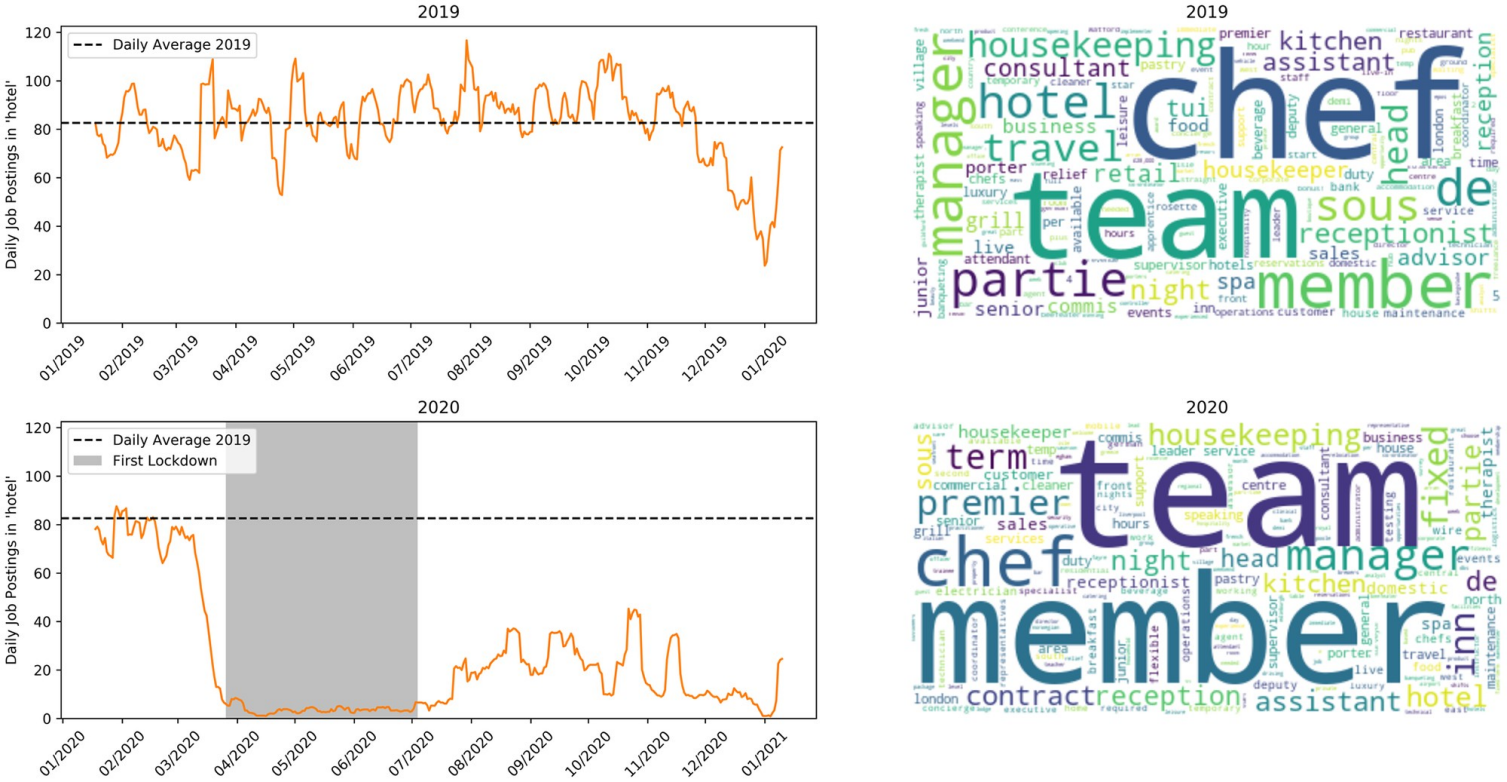
Jobs matching ‘hotel’. Top row is 2019, bottom is 2020.

The time series shown in Fig 4 shows jobs in the ‘hotel’ class. This collects a number of service industry jobs in the hotel and hospitality industry. There is a complete collapse in the number of vacancies in this sector in the wake of the COVID-19 crisis with the drop beginning the week prior to the first major announcement of lockdown restrictions. The number of vacancies remained low throughout lockdown and in the weeks after. A recovery began in mid-August, summer holiday season, but vacancy postings remain at less than half of pre-crisis levels. Interestingly ‘fixed term’ is visible in the word cloud for 2020, indicating more non-permanent jobs being advertised.

**Fig 5:**
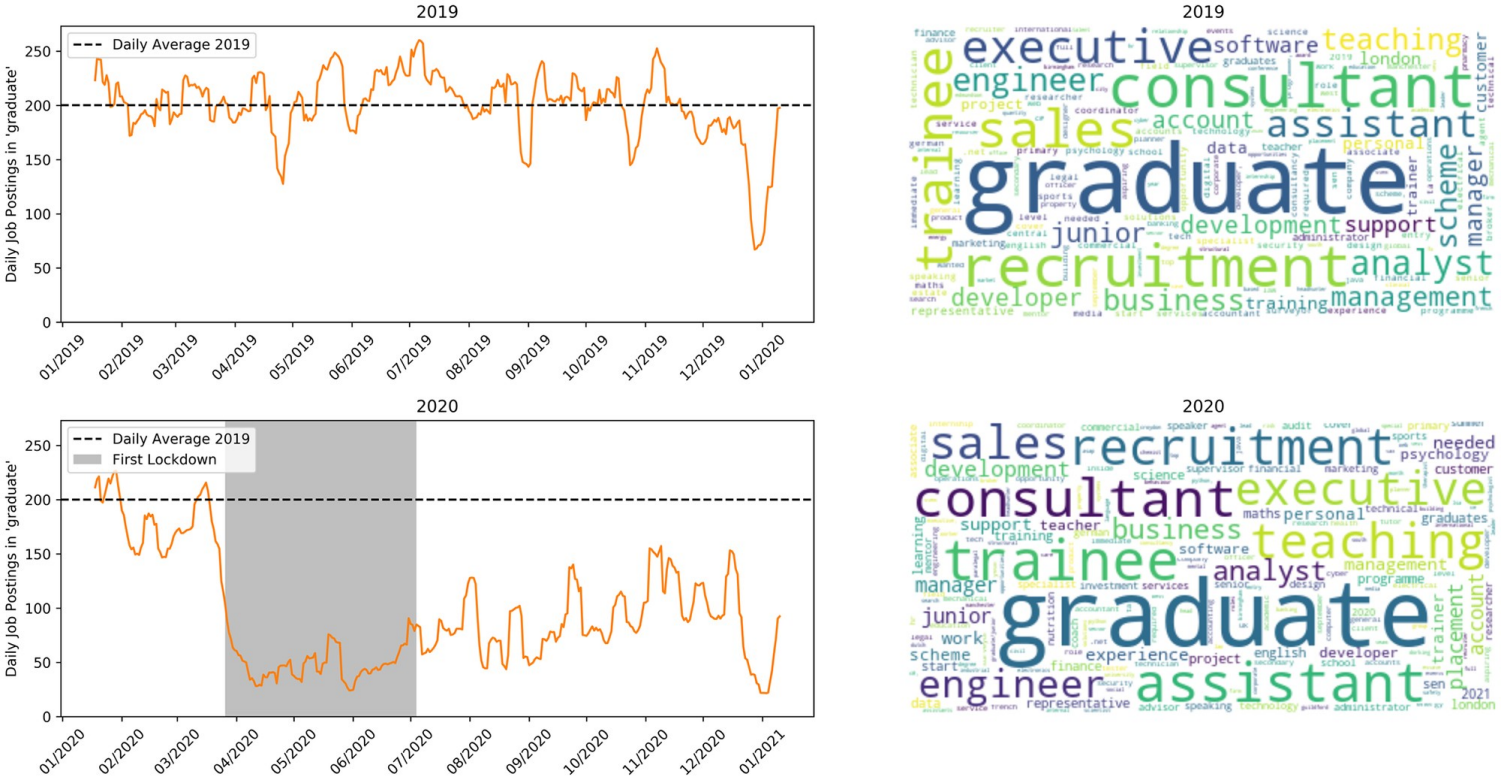
Jobs matching ‘graduate’. Top row is 2019, bottom is 2020.

Fig 5 shows ‘graduate’ jobs, i.e., vacancies for graduates entering the workforce via job training or recruitment schemes. There is a drastic drop in the number of adverts for these schemes, with a very slow recovery in the weeks after lockdown.

**Fig 6:**
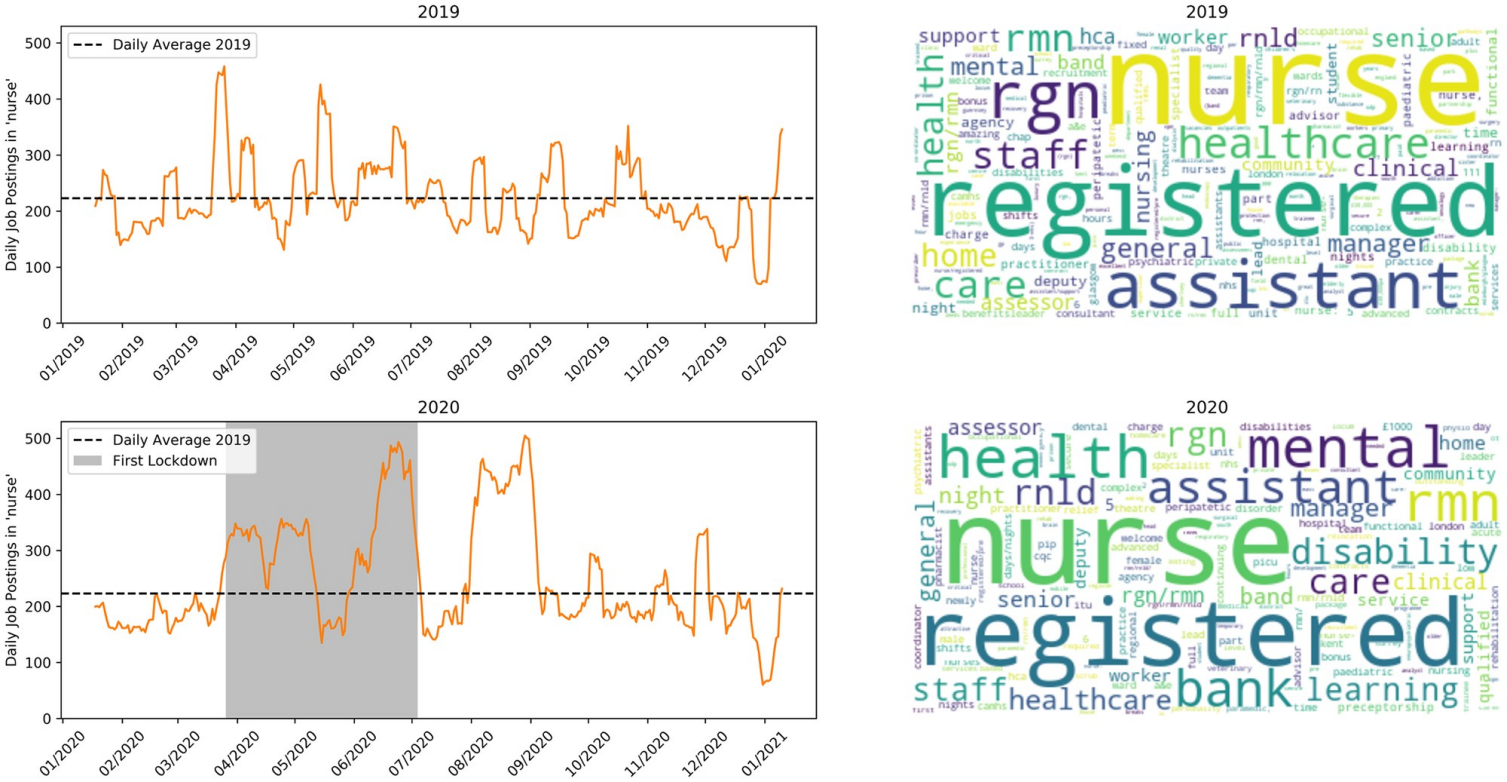
Jobs matching ‘nurse’. Top row is 2019, bottom is 2020.

Fig 6 shows nursing jobs. As might have been expected during a public health emergency, the number of ads for nurses increased somewhat during lockdown and in the weeks after. The word cloud for 2020 shows a high occurrence of the terms ‘mental health’. The mental health implications of COVID-19 have been much discussed [44] and hiring decisions are reflecting this.

**Fig 7:**
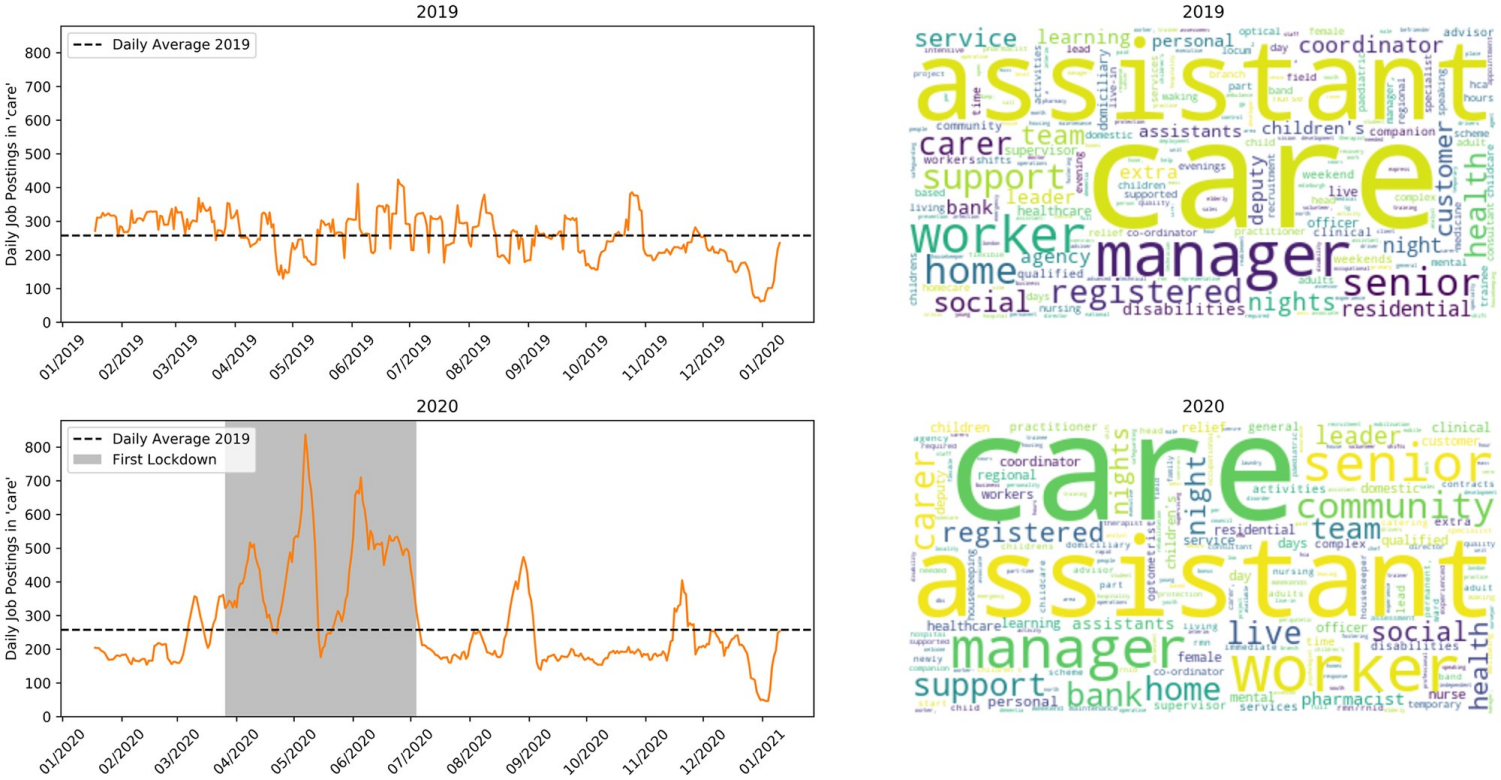
Jobs matching ‘care’. Top row is 2019, bottom is 2020.

Fig 7 shows care work jobs. This time series also shows an increased demand for care workers during the crisis, especially during the lockdown period. The closure of many public services through this period likely increases the need that older or disabled people have for special assistance.

**Fig 8:**
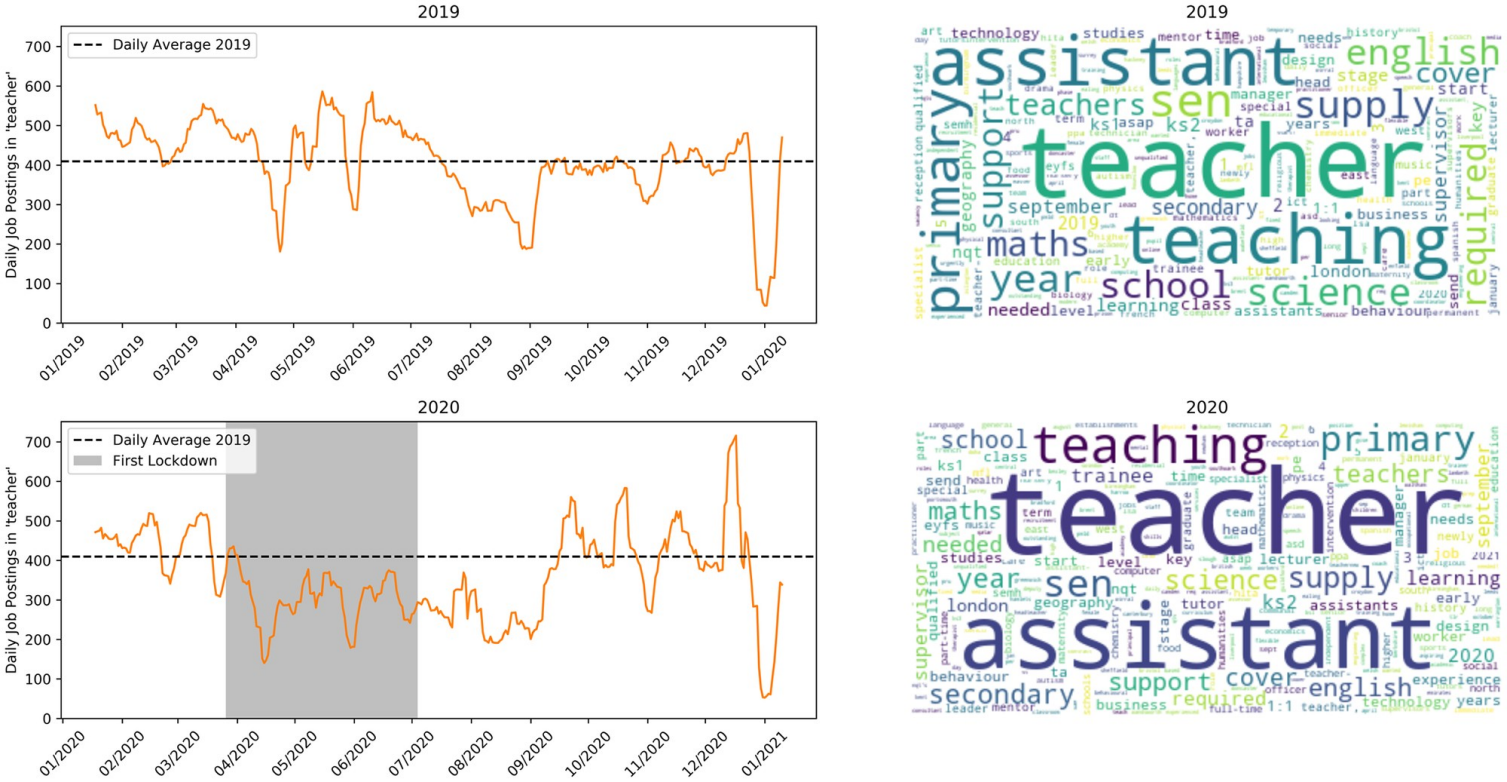
Jobs matching ‘teacher’. Top row is 2019, bottom is 2020.

Fig 8 shows teaching jobs. This time series shows a slight decrease in the demand for teachers during lockdown, however some of the backlog seems to have been filled once the first easing of restrictions was announced. After lockdown the number of vacancies in this sector is still down slightly, but is reduced far less than other sectors and the average.

**Fig 9:**
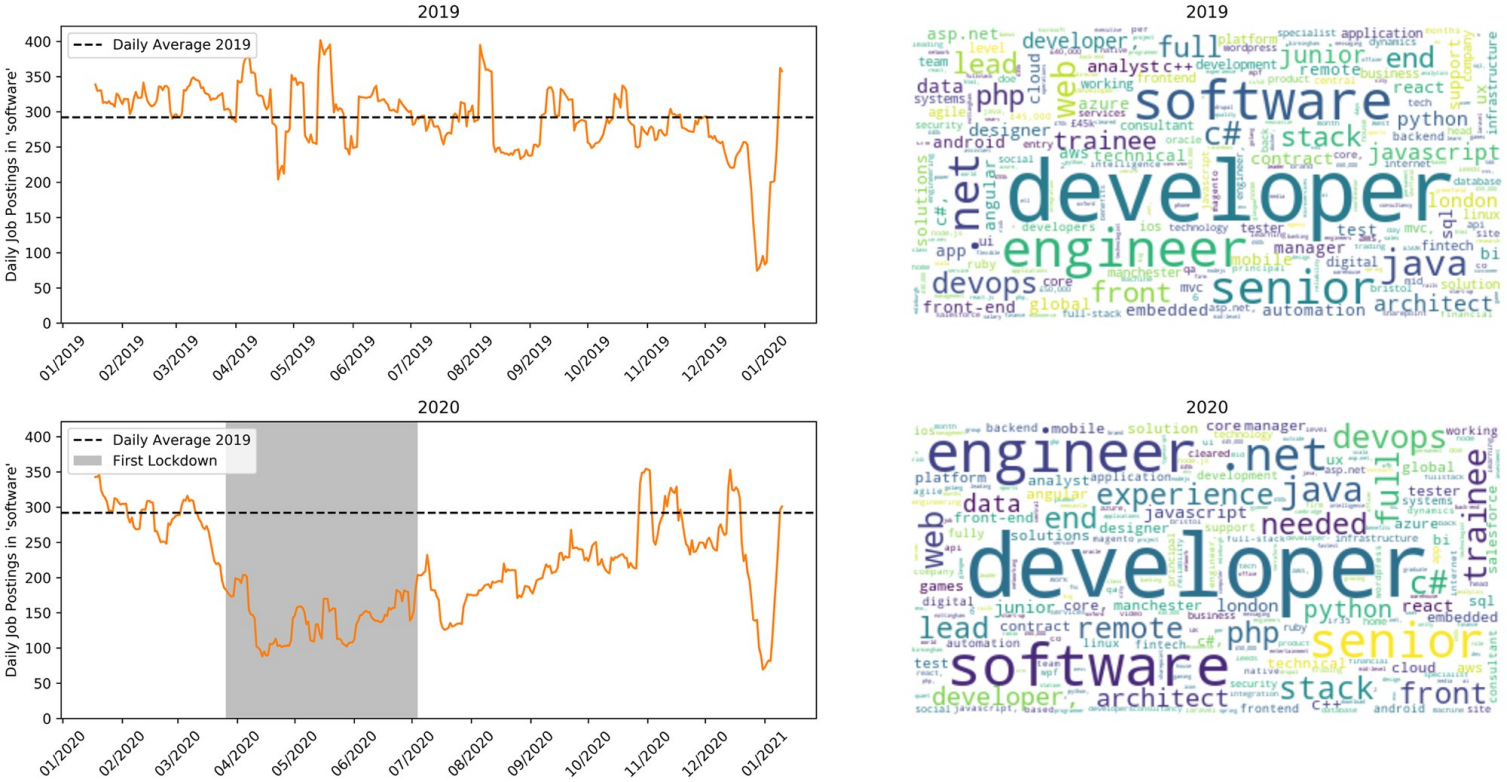
Jobs matching ‘software’. Top row is 2019, bottom is 2020.

Fig 9 shows software jobs. The number of these vacancies is went down significantly during lockdown, with the post-lockdown demonstrating a slow recovery. Many software development jobs can, in principle, be done remotely but the wider economic uncertainty likely affects the number of vacancies companies are willing to advertise. The term ‘remote’ is visible in the 2020 word cloud, an indication that remote working in this industry is becoming widespread.

**Fig 10:**
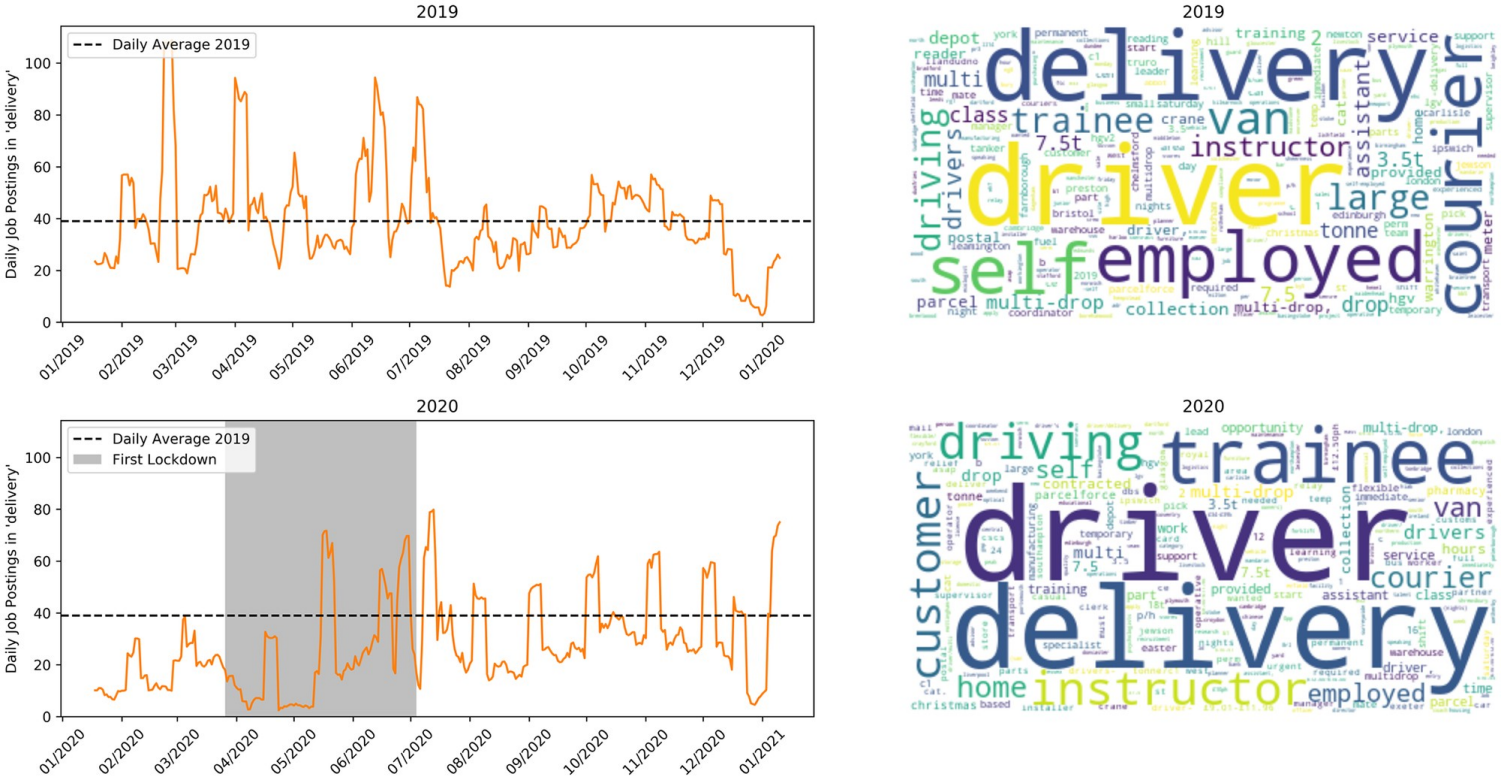
Jobs matching ‘delivery’. Top row is 2019, bottom is 2020.

Finally Fig 10 shows delivery jobs. With most high street stores closed throughout lockdown the number of deliveries could reasonably be expected to increase. However, at the beginning of lockdown the number of adverts for drivers decreased significantly, in line with trends in other sectors. With the easing of restrictions some of this backlog was filled and the number of adverts is now around (though slightly below) pre-crisis levels. This unexpected behaviour shows that companies reacted to lockdown by reducing costs, rather than hiring in anticipation of increased demand. In late 2020 the spikes at the end of every month starting in August are 200 identical adverts for trainee driving instructors that appear to have been misclassified as they contain many terms relating to driving. The total number of delivery jobs is very low, it is therefore likely that Reed is not a popular platform to advertise this type of job.

## Jobs by location

The regions of the UK were not affected equally by COVID-19. As of January 27th, 2021 the death rates per 100000 in the North East and North West were 198.7 and 203.4 respectively, while the rate in the South West was 89.6 [1]. After the first lockdown in some areas with high case rates a local lockdown was imposed. The first of these was the city of Leicester and surrounding areas on July 4th, 2020 [9], with another significant local lockdown implemented on July 25th, 2020 in the area of Blackburn with Darwen [8]. Large areas of the North of England were subsequently subjected to more severe restrictions than the rest of the UK [45].

**Fig 11:**
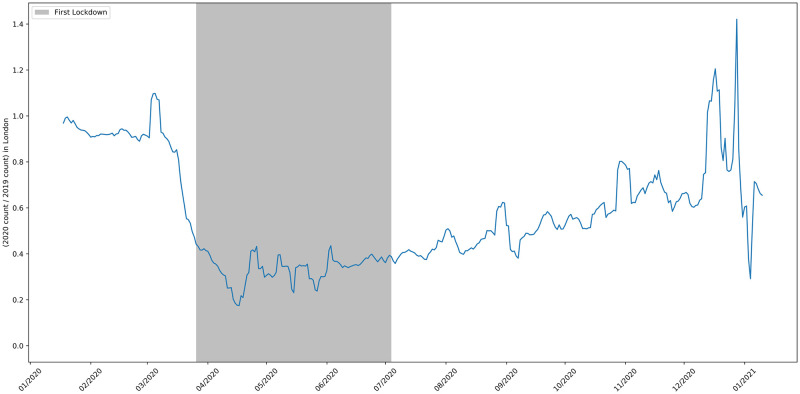
The 2020 to 2019 ratio of job adverts in the Greater London area.

**Fig 12:**
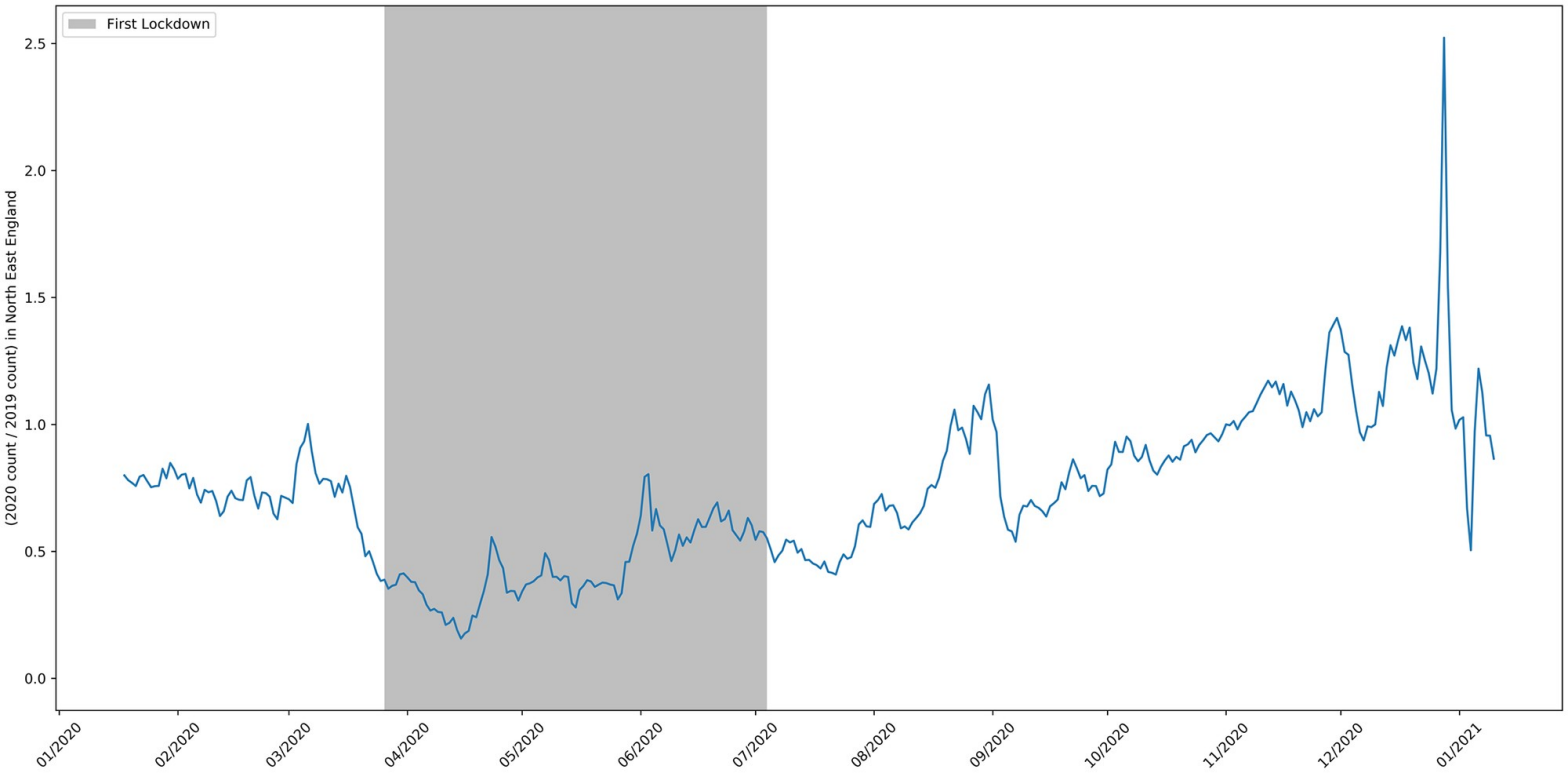
The 2020 to 2019 ratio of job adverts in the North West.

**Fig 13:**
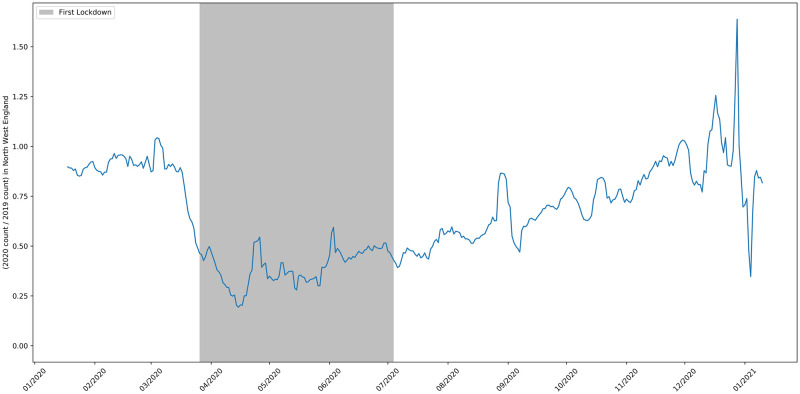
The 2020 to 2019 ratio of job adverts in the South West.

**Fig 14:**
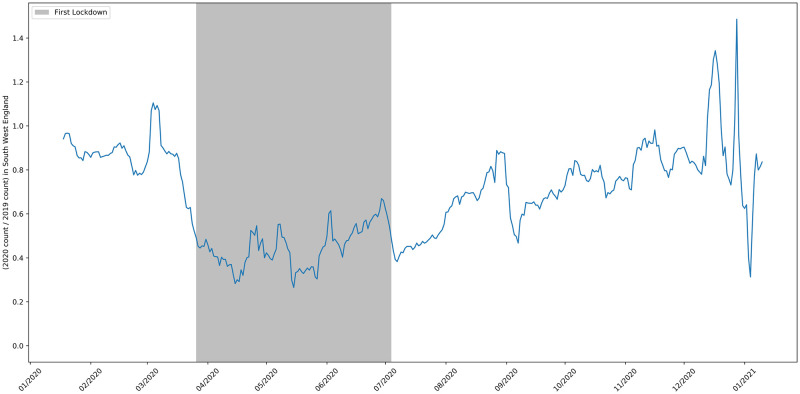
The ratio of job adverts in the North East.

**Fig 15:**
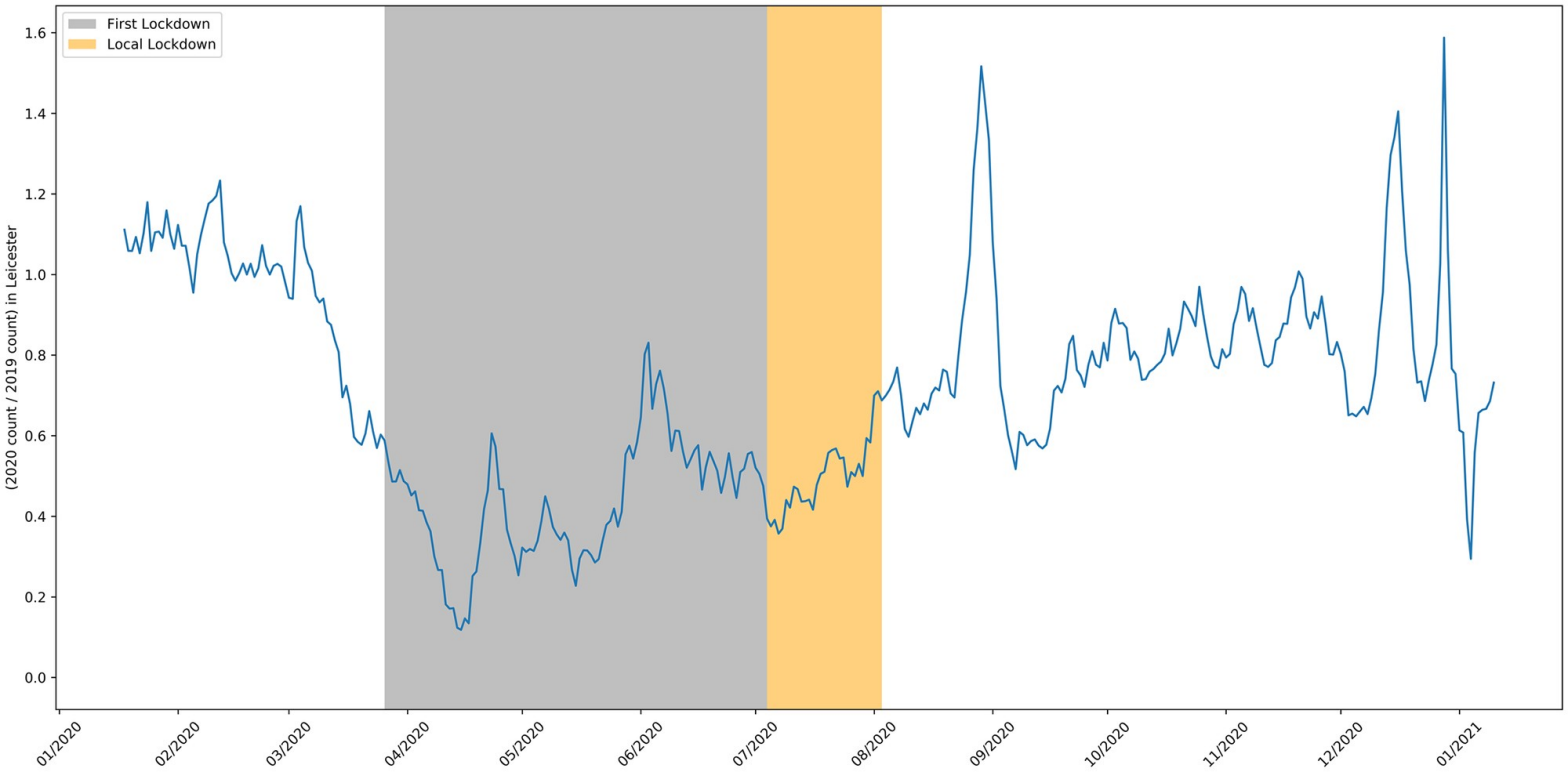
The 2020 to 2019 ratio of job adverts in the city of Leicester.

**Fig 16:**
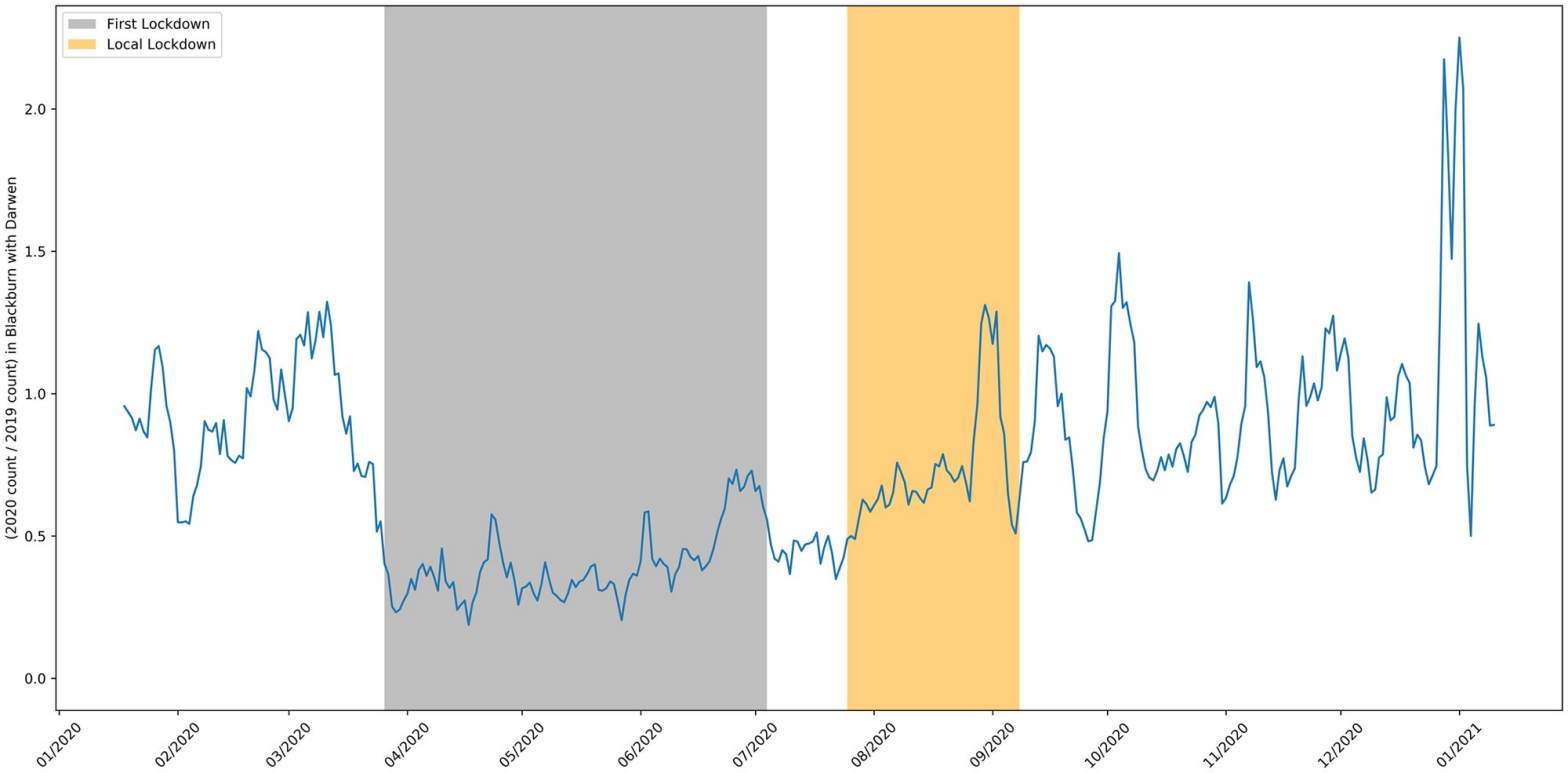
The 2020 to 2019 ratio of job adverts in area of Blackburn with Darwen.

This section will examine how the effect of the crisis on vacancies was distributed across the UK; comparing regions which were affected to different degrees by COVID-19 and examining if local lockdowns have a compounding effect on depressing the job vacancy data. Figs 11–16 show the ratio of the 7 day running average in 2020 compared to 2019. 2020 is a leap year, so has one more day than 2019, for simplicity, this day is skipped in the figures in this section.

Fig 11 shows the ratio of job advert counts in London in 2020 compared to 2019. The pattern for London reflects the pattern for the whole UK shown in Fig 2. The North West was the worst affected region in the UK and subject to different rules after August 5th [45]. Despite this, the time series in Fig 12 looks broadly similar to the one for Greater London and the UK as a whole. The South West was the region of England least affected by COVID-19. Fig 13 shows that by mid-September the number of job vacancies here had recovered slightly more than the UK average, but the drop in job adverts and the slow recovery after lockdown easing reflects the pattern for the whole of the UK.

The north east was one of the worst affected regions in the UK. Surprisingly, unlike the pattern for the whole of the UK and the other regions, Fig 14 shows that after the lockdown eased the number of job adverts recovered and exceeded pre-crisis levels after mid-August. However, note that the absolute number of adverts for this area is significantly lower, at around 200 postings per day, so the variance of the signal is higher.

The first local lockdown, where the rules of the national lockdown period were extended, was implemented within the city of Leicester and surrounding areas [9]. Fig 15 shows there is no marked change in the number of job adverts in response to this local restriction, with the count staying around its previous (depressed) level. Lifting the local restrictions does correspond to an increase in the number of adverts posted, but this increase is slight and in line with national trends, so determining causality requires further investigation.

The story for Blackburn with Darwen in Fig 16 is similar to Leicester. There is a suggestion that job vacancies increased after the local lockdown lifted. However trends are in line with the rest of the UK and Blackburn is in the north of England and remained subject to different rules [45] after the local lockdown ended.

## Salary and contract type

Other interesting information associated with job adverts includes the salary, contract type (temporary, permanent or contract), and mode of employment (full time or part time). In this section I investigate if there has been any change in the frequency of different contract types, modes of employment, or distribution of salaries. The roughly 9 month periods from March 16th, 2019 to January 10th, 2020 and March 16th, 2020 to January 10th, 2021 are compared, where the second period encompasses the COVID ‘shock’ to the job market.

**Fig 17:**
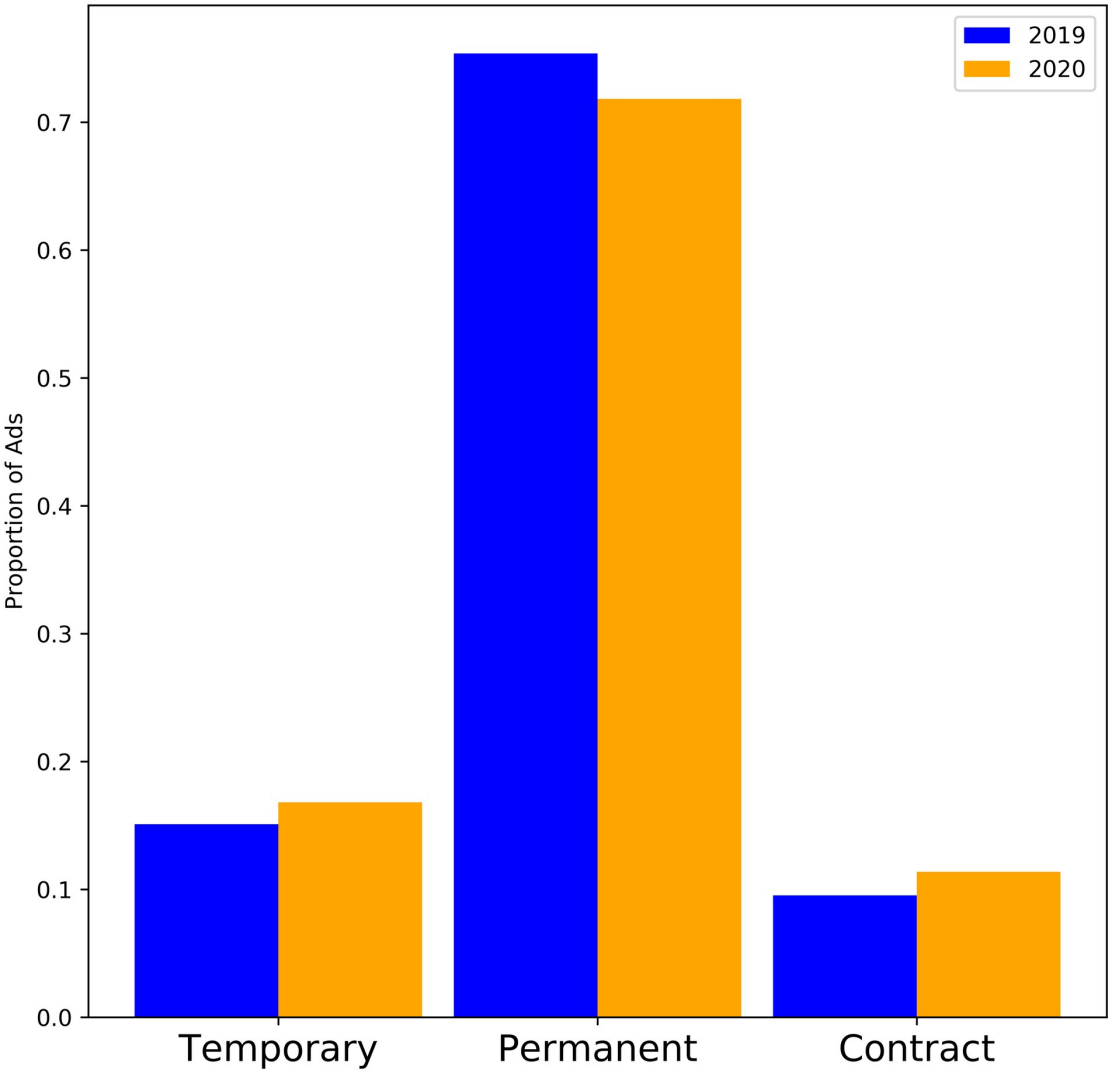
Proportion of job ads corresponding to three types of contract. **Before Lockdown**: Temporary: 15%, Permanent: 75%, Contract 10%. **After Lockdown**: Temporary: 17%, Permanent: 72%, Contract 11%.

**Fig 18:**
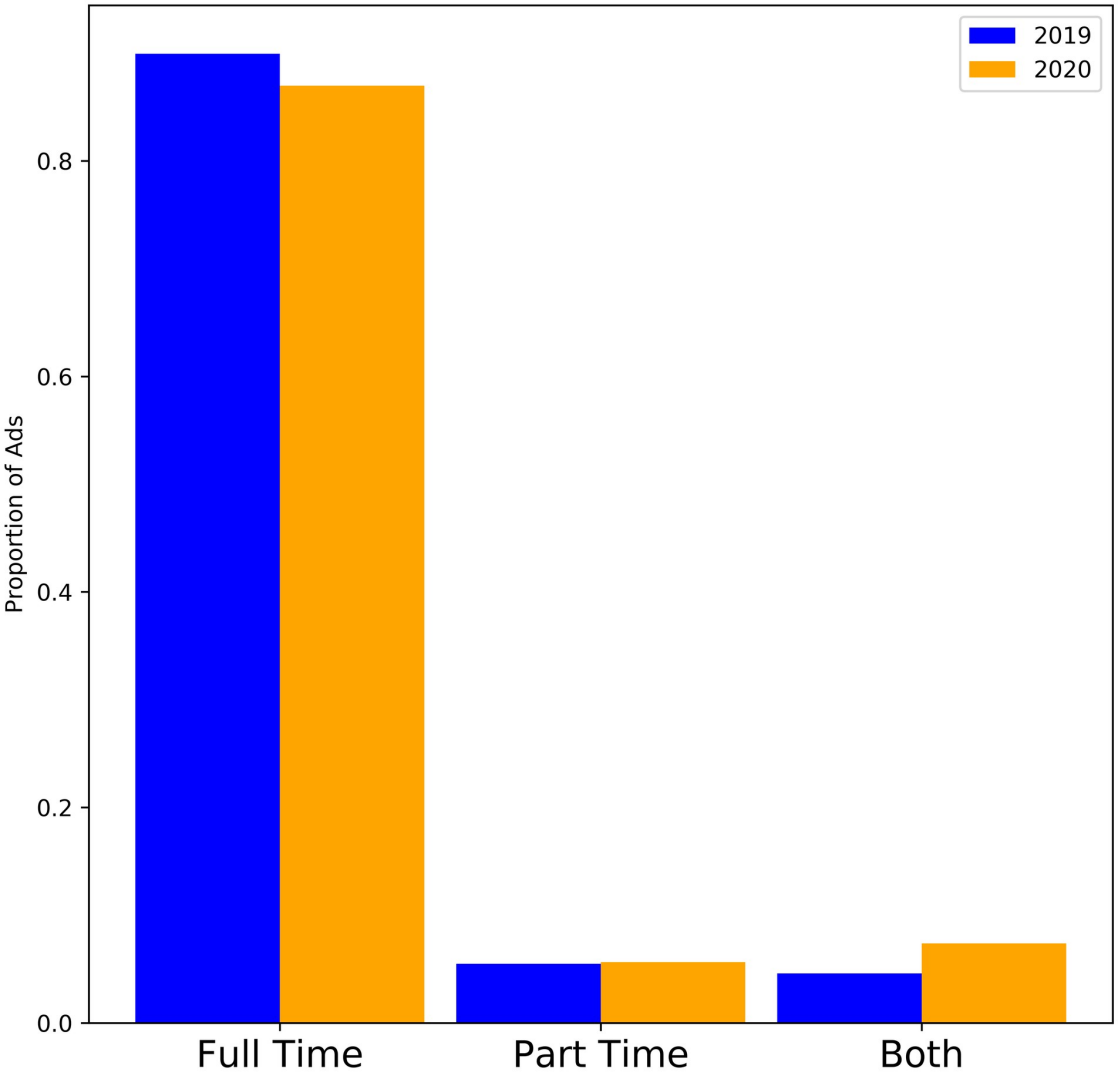
Proportion of job ads corresponding to full time and part time employment. **Before Lockdown**: Full Time: 90%, Part Time: 5%, Both: 5%. **After Lockdown**: Full Time: 87%, Part Time: 6%, Both: 7%.

**Fig 19:**
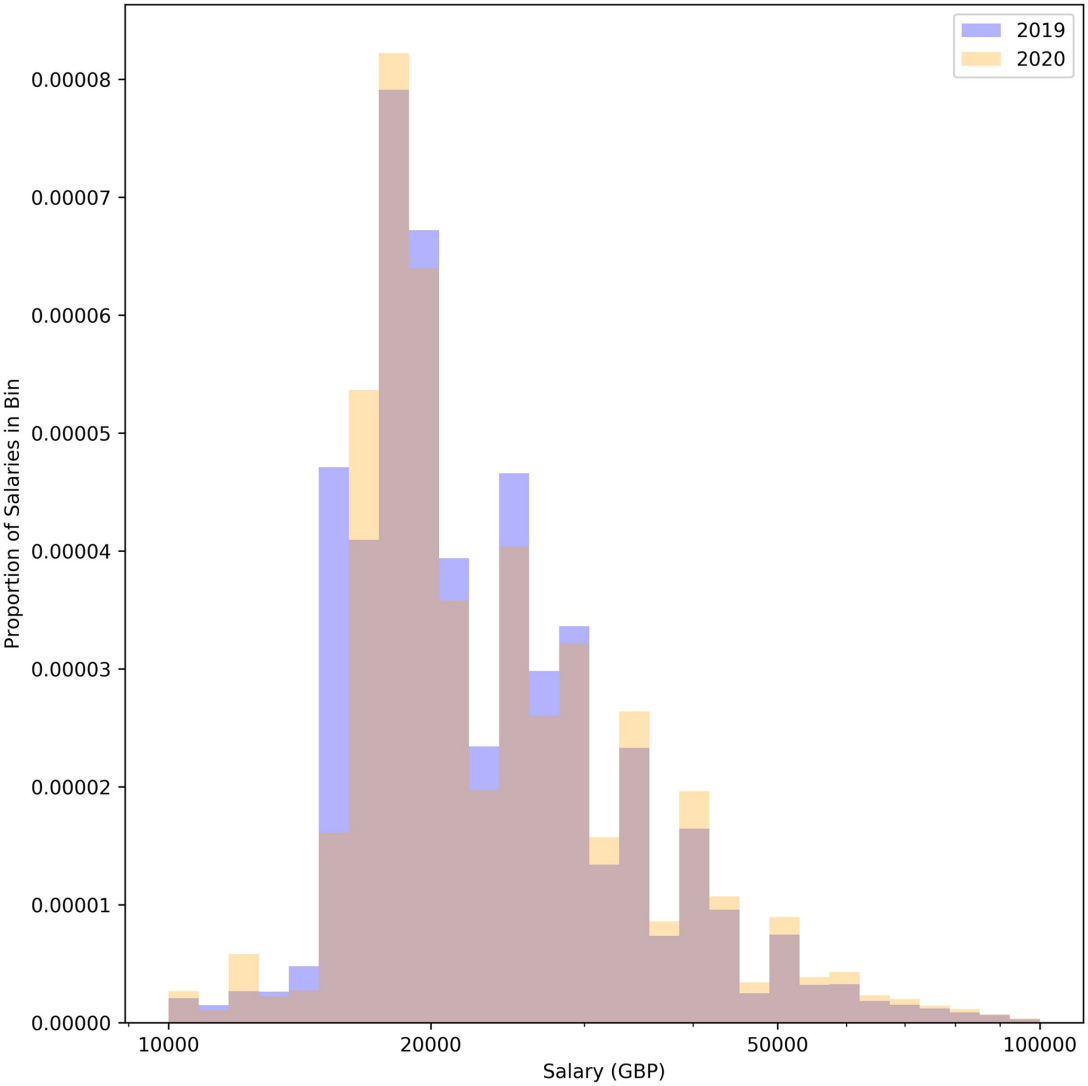
Distribution of annual salaries (obtained from the ‘yearly minimum salary’ field in the JSON returned by the Reed API). **2019**: Mean Salary: 31285 GBP, Median Salary: 25000 GBP. **2020**: Mean Salary: 31645 GBP, Median Salary: 25714 GBP.

Fig 17 shows the proportion of jobs advertised as temporary, permanent, or contract. Fig 18 shows the proportion of jobs advertised as full time or part time. Fig 19 shows the distribution of advertised salaries. There is a very small increase in mean and median annual salary. A t-test comparing the means returns a p-value of 0.167 (no significant difference) while a 2 sample KS-test returns a highly significant (p-value <10^−10^) difference between the two distributions. Using inflation data from [46] to adjust the monthly salaries to a common baseline yields similar results (t-test p-value 0.75, KS-test p-value <10^−10^) and visually indistinguishable histograms. There is also a slight but significant (*χ*^2^ test p-value <10^−10^) trend towards part time and non-permanent jobs.

## Web interface

The topic and geographic filters can be combined to look at, e.g., ‘teaching jobs in the south west’ and the salary and contract type analysis can be performed on a sectoral or geographic level. The most effective way to enable stakeholders, like labour market analysts or local authorities, to get this information is likely the creation of an interactive dashboard using the methodology and data described in this work.

**Fig 20:**
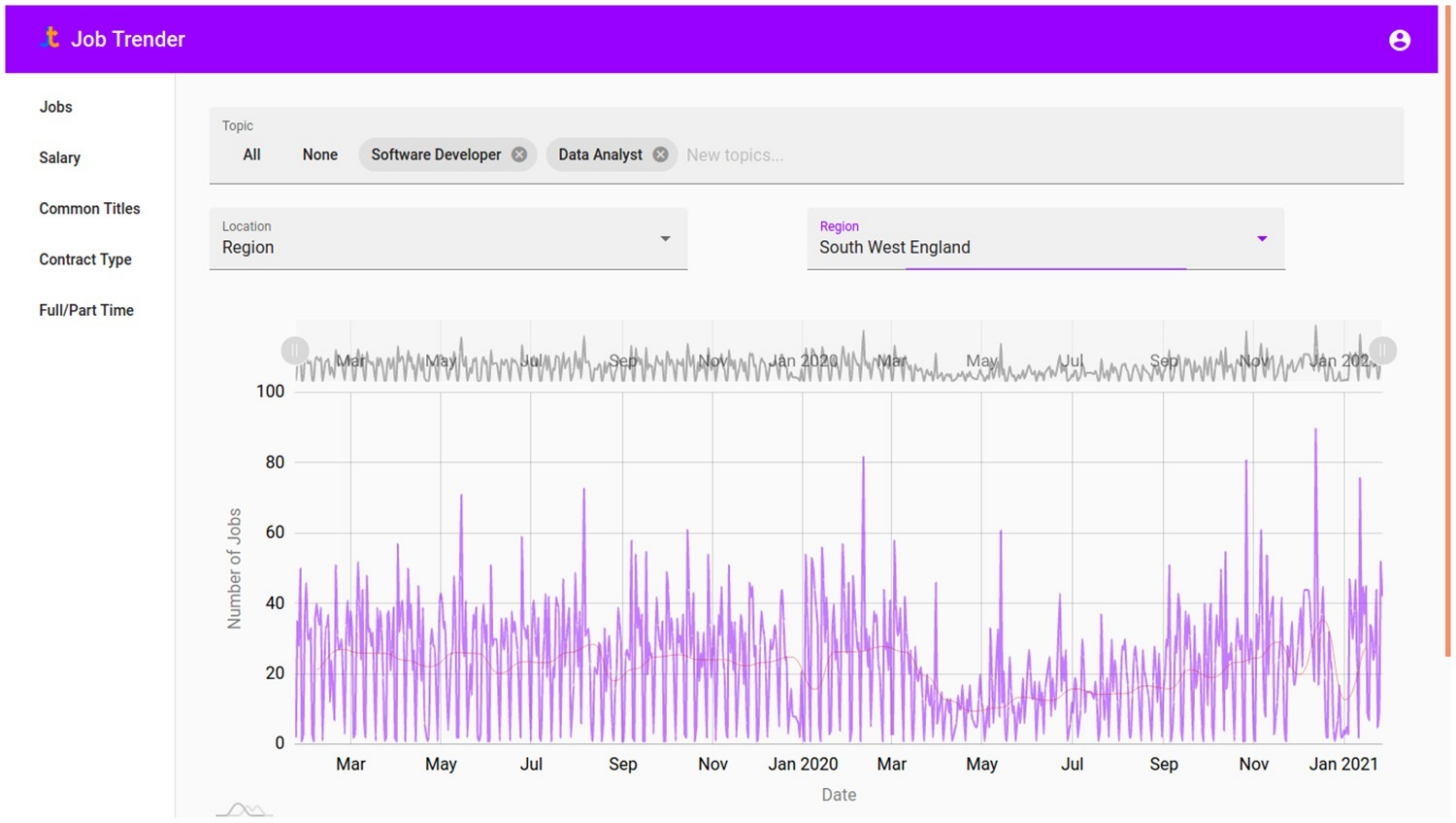
Job ads visualised with the online tool. Shown here are software and data jobs in the South West of England over the entire collection period.

I have created such a dashboard and made it available online at https://jobtrender.com/. On the ‘back end’ the methods described in this work are used to classify and locate job ads. These are then queried through a simple search interface, as shown in Fig 20.

## Discussion

The data tells us that companies have responded to the COVID-19 crisis by reducing hiring significantly. The global patterns agree with the findings of [22] and Indeed, however the use of a full year of data gives a clearer view of the situation and extent of recovery. Certain sectors, like hospitality and graduate recruitment, have been particularly affected while others, like care work and teaching recruitment, have not been negatively impacted. The conditions of work in a post COVID-19 world, at least in terms of contracts, hours of employment and salary seem to be broadly unchanged but with a very small but statistically significant shift towards higher salary, non-permanent and part time work. One hypothesis is that the lower paying jobs aren’t being advertised, increasing the median salary, while the jobs that are being advertised are shifting towards part time and fixed contracts, as companies try to hedge against uncertainty. There may also be seasonal and long term trends. A full understanding of this pattern requires more data.

Regional differences are not as strong as sectoral ones, mirroring the results obtained in the US by [19]. The North East seems to have recovered faster than the national average, but the absolute number of adverts in this area is much lower than in other regions. This could be due to a number of factors: a previously depressed local job market; the local (un)popularity of Reed, or even that the types of jobs available in this region not being openly advertised online. Further investigation is required before any strong conclusions can be drawn. It also seems that local lockdowns do not have a compounding effect on the job market but may postpone recovery, again more investigation is required to fully support this conclusion.

Future work could look at improving our topic detection algorithms, for example using a semi-supervised approach to detect known sectors [47]. We could also perform topic detection within sectors to study changes in job descriptions in response to new conditions of work e.g. an increase in home working [48].

Job adverts alone do not present a complete view of the national job market. Job boards typically do not capture data on recall hiring [49], the temporary layoff and rehiring of the same individuals, often with worse terms for the employee [50]. This practice has been prevalent in the US [51] and could have occurred in the UK, signalled by the large umber of new unemployment claims at the start of the crisis [52]. However the UK’s Coronavirus Job Retention Scheme [15] was established early on, backdated to March 1st, 2020 and is currently in place until September, 2021. It offers monthly grants to employers to pay 80% of staff wage and employment costs. Due to this scheme the effect of recall hiring may be smaller in the UK than the US, but it cannot be estimated from our data.

Job vacancy data tells us about the amount of hiring but not firing. The net result (new hires minus terminations) would give the full picture on jobs in the economy or any particular sector. An apparent recovery to pre-pandemic levels of vacancy advertising in a sector does not indicate a full recovery if that sector experienced widespread job loss. The Coronavirus Job Retention Scheme aimed to prevent this, but cannot be assumed to be completely successful. Job vacancy data can only give a partial view of the degree of decline or recovery, a complete picture requires combining data sources. This is another reason why open data sets such as this one are crucial.

There are also a number of technical issues with how job boards are used. For example, some job adverts refer to multiple open positions; some job adverts may be reposted after failing to recruit anyone; adverts may be posted and later withdrawn, though still collected by our API call. It is also unknown how this behaviour has changed due to the COVID crisis. There may be a larger number of withdrawn ads (especially around March, 2020) but fewer reposted ads as vacancies are filled more easily. As mentioned in the Introduction we did not see any notable change in number of withdrawn vacancies (valid post ids with null data) across the study period. Regarding reposted ads, often multiple copies of the same advert are posted by recruitment agencies which account for a large proportion of repeated adverts. This makes it difficult to identify been reposted after failing to be filled. Counting the number of job adverts which exactly match at least one other advert, in 2019 this was roughly 9% of all adverts while in 2020 it was 8%. The job advert data analysed here does not indicate a marked change in withdrawals or reposts, more analysis, and possibly additional data, is needed to fully explore these issues, but that is beyond the scope of this work.

Finally, throughout this work all job adverts come from a single source. Though Reed is a large job board there are several others which are as popular in the UK, though they do not allow data collection through an open API. There are also many specialist recruitment websites e.g. jobs.ac.uk for academic jobs as well as local ones e.g. devonjobs.gov.uk. Accumulating job advert information from all of these sources would remove many of the biases (known and unknown) introduced by relying on a single source of data. For example, the EU Center for the Development of Vocational Training [53] has attempted to collect online job adverts from a variety of sources across Europe, though they do not reshare their data. What we can do with a single data source is look at *relative* changes in the number of postings, which makes this an effective method for studying the impact of shocks like COVID.

The research of [30] as well as the company Burning Glass shows that using machine learning and geo-inference techniques on job ads can help us to understand labour market trends and skills demand. This research extends those methods and shows they can be used to study short term shocks to the labour market, such as COVID-19 and associated lockdown policies. This is a simple and transparent way to study the effect of economic shocks and major government interventions on labour market activity across employment sectors and geographies. This is complementary data to e.g. survey methodology [17] and is accessible to academics, unlike the very useful but opaque methodology of [19] and Burning Glass.

Though ‘evidence based policy’ is something of a catchphrase [54], it is of course desirable that policy makers consider all the available information before taking action. The effect on the job market should be considered when extending or relaxing lockdown rules and especially in relation to extending or ending furlough and other compensation schemes. We hope that this paper and its methods provide valuable insights for broad or targeted policy interventions.
